# Activation of insulin-like growth factor 1 receptor in patients with non-small cell lung cancer

**DOI:** 10.18632/oncotarget.3796

**Published:** 2015-04-12

**Authors:** Jin-Soo Kim, Edward S. Kim, Diane Liu, J. Jack Lee, Carmen Behrens, Scott M. Lippman, Waun Ki Hong, Ignacio I. Wistuba, Euni Lee, Ho-Young Lee

**Affiliations:** ^1^ Department of Thoracic/Head & Neck Medical Oncology, The University of Texas MD Anderson Cancer Center, Houston, TX, USA; ^2^ Department of Biostatistics, The University of Texas MD Anderson Cancer Center, Houston, TX, USA; ^3^ Department of Pathology, The University of Texas MD Anderson Cancer Center, Houston, TX, USA; ^4^ College of Pharmacy and Research Institute of Pharmaceutical Sciences, Seoul National University, Seoul, Republic of Korea; ^5^ Present address: Department of Internal Medicine, Seoul National University Boramae Medical Center, Seoul, Republic of Korea

**Keywords:** non-small cell lung cancer, insulin-like growth factor-1 receptor, prognosis, tissue microarray

## Abstract

According to previous reports demonstrating the implication of insulin-like growth factor receptor (IGF-1R) signaling in non-small cell lung cancer (NSCLC), in this study, the potential prognostic values of IGF-1R expression/activation were analyzed. The expression and activation of IGF-1R were evaluated in two tissue microarray (TMA) sets from NSCLC patients (*N* = 352 for TMA I, and *N* = 353 for TMA II). Alterations in IGF-1R protein or mRNA expression in NSCLC patients were evaluated using publicly available data from The Cancer Genome Atlas (TCGA). We found that membranous and cytoplasmic IGF-1R expressions were significantly associated with squamous cell carcinoma (SCC) in both of the TMAs. Analysis of the TCGA data revealed increased mRNA levels in NSCLC patients, which was significantly associated with reductions in overall survival (OS) (median survival 26.51 vs. 47.77 months, *P* = 0.017) and disease-free survival (median survival 17.44 vs. 37.65 months, *P* = 0.045) only in NSCLC patients with adenocarcinoma (ADC). These data suggest that IGF-1R is activated in patients with NSCLC, particularly those with SCC. IGF-1R mRNA expression is a potential prognostic factor in patients with NSCLC, especially those with ADC. Further studies are warranted to investigate the prognostic value of IGF-1R in NSCLC patients.

## INTRODUCTION

Lung cancer is the leading cause of cancer deaths and results in more than one million deaths worldwide annually [1]. Approximately 150,000 deaths occur in the USA annually [2], with an estimated 16.8% of patients surviving 5 years or longer. While rates of new cases and deaths have fallen steadily in recent years, these declines have been slow and small in magnitude compared with other cancers. Therefore, the prevention and treatment of lung cancer remain unmet needs. However, chemoprevention trials for lung cancer have yielded negative results [3], and the currently available protocols for lung cancer treatment including conventional chemotherapy, radiotherapy, and molecularly targeted therapy appear to have minimal benefits for improving the survival of lung cancer patients [4]. The identification of key biomarkers with prognostic value would help guide future clinical trials of lung cancer therapies.

Type 1 insulin-like growth factor receptor (IGF-1R), a membrane-associated receptor tyrosine kinase, plays a clear role in cell proliferation, survival, transformation, angiogenesis, and invasion [5]. Deregulation of IGF signaling has been described in several cancer types, including both non-small cell lung cancer (NSCLC) and small cell lung cancer (SCLC) [6], and high expression of IGF-1R in NSCLC has been reported [7, 8]. The therapeutic value of an IGF-1R antibody has also been assessed in recent trials. Figitumumab, which targets IGF-1R, initially exhibited promising efficacy against advanced NSCLC in a phase II study [9], but failed to exhibit safety and efficacy in a phase III trial, and further clinical development was stopped [10]. Furthermore, preclinical and clinical studies do not consistently support the predictive or prognostic value of IGF-1R expression in NSCLC [7, 11-13].

Because previous studies have reported a potential role of IGF-1R pathways in lung cancer [14], we analyzed the expression of phospho-IGF-1R and IGF-1R in NSCLC biopsy samples using two large repositories of tissues annotated for relevant histological and clinical variables. We then assessed the relationship of these markers with lung cancer survival. In addition, alterations in IGF-1R mRNA and protein expression and their relationships with overall survival (OS) and disease-free survival (DFS) were evaluated using data from The Cancer Genome Atlas (TCGA).

## RESULTS

### Expression of IGF-1R and pIGF-1R

**Figure 1:**
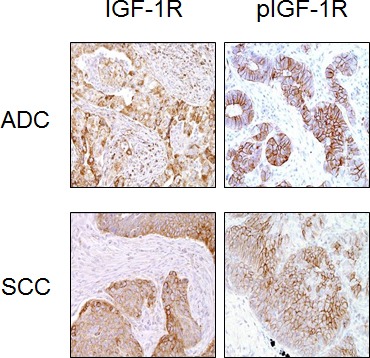
Representative photomicrographs of the immunohistochemical analysis of IGF-1R and phospho-IGF-1R protein expression in histological tissue sections of adenocarcinoma (ADC) and squamous cell carcinoma (SCC)

**Figure 2:**
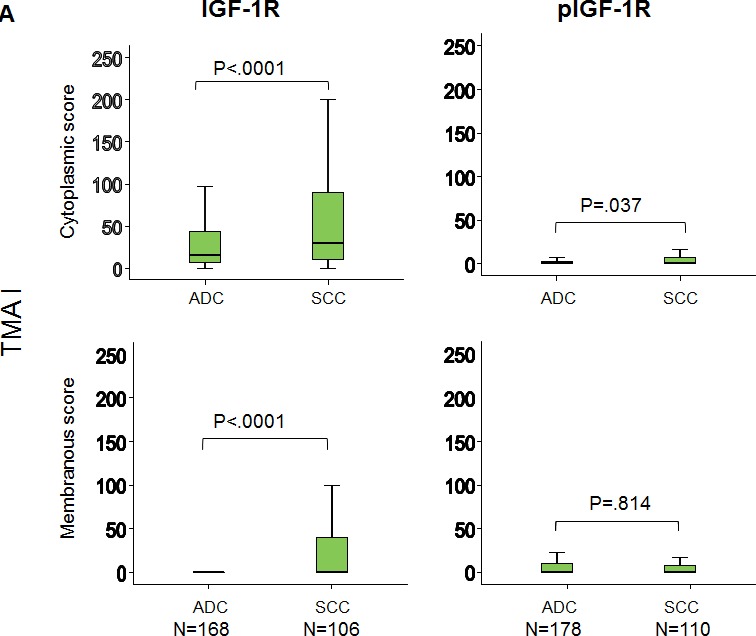

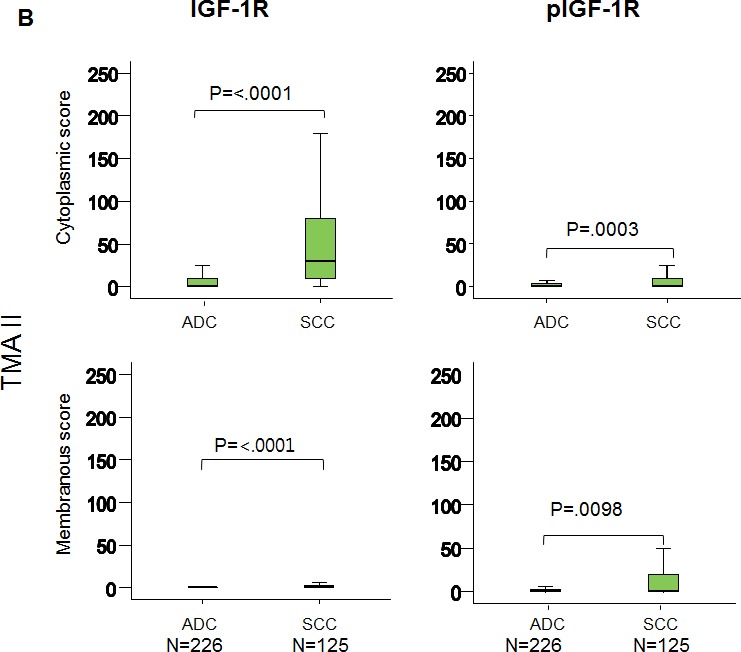
Increased expression of IGF-1R and phospho-IGF-1R in SCC compared to ADC (**A**) TMA I and (**B**) TMA II. ADC: adenocarcinoma, SCC: squamous cell carcinoma. P-values were calculated using the Mann–Whitney test.

We performed an IHC analysis of IGF-1R and pIGF-1R (Y1135/36) expression using two TMAs. Expression levels of cytoplasmic and membranous location were well-correlated for each marker. Representative IHC results are shown in Fig. 1. Similar to previous findings, IGF-1R expression was primarily cytoplasmic (Figs. 2A and 2B) and significantly higher in patients with SCC compared to ADC in both TMAs (Figs. 2A and 2B). The cytoplasmic expression levels of pIGF-1R were also significantly higher in patients with SCC than ADC in both of the TMAs, while the membranous pIGF-1R expression level was significantly associated with SCC only in TMA II (Figs. 2A and 2B). The expression levels of the biomarker were not significantly different between stages (data not shown).

**Table 1:** Patients characteristics in tissue microarray (TMA) dataset I

Characteristic	TMA I (N=352)
Median age	
Years (Range)	66 (34-90)
Gender	
Female	189 (53.7%)
Male	160 (45.5%)
Histology	
Adenocarcinoma	231 (65.6%)
Squamous cell carcinoma	121 (34.3%)
Ethnicity	
Caucasian	313 (88.9%)
Asian	14 (4.0%)
African American	15 (4.3%)
Hispanic	10 (2.8%)
Smoking status*	
Never	63 (17.9%)
Former	174 (49.4%)
Current	114 (32.4%)
Unknown	1 (0.3%)
Pathological T stage^†^	
T1	130 (36.9%)
T2	185 (52.6%)
T3	18 (5.1%)
T4	20 (5.7%)
Pathological N stage^†^	
N0	253 (71.9%)
N1	60 (17.0%)
N2	39 (11.1%)
Nx	
Final Stage^†^	
I	225 (63.9%)
II	65 (18.5%)
III	50 (14.2%)
IV	12 (3.4%)

* Smoking history was assigned based on the CDC definitions (Accessed on Jun 29, 2010. http://www.cdc.gov/nchs/nhis/tobacco/tobacco_glossary.htm)

Never smoker: An adult who has never smoked, or who has smoked less than 100 cigarettes in his or her lifetime. Former smoker: An adult who has smoked at least 100 cigarettes in his or her lifetime but who had quit smoking at the time of interview. Current smoker: An adult who has smoked 100 cigarettes in his or her lifetime and who currently smokes cigarettes.

† According to the American Joint Committee on Cancer Staging Manual 6th edition.

**Table 2:** Patients characteristics in tissue microarray (TMA) dataset II

Characteristic	TMA II (N=353)
Median age	
Years (Range)	66 (34-90)
Gender	
Female	174 (49.3%)
Male	179 (50.7%)
Histology	
Adenocarcinoma	227 (64.3%)
Squamous cell carcinoma	126 (35.7%)
Ethnicity	
Caucasian	316 (89.5%)
Asian	5(1.4%)
African American	18 (5.1%)
Hispanic	14 (4%)
Smoking status*	
Never	38 (10.8%)
Former	164 (46.5%)
Current	151 (42.8%)
Unknown	
Pathological T stage^†^	
T1	131(37.1%)
T2	202(57.2%)
T3	20(5.7%)
Pathological N stage^†^	
N0	236 (67.6%)
N1	66 (18.9%)
N2	47 (13.5%)
Final Stage^†^	
IA	100 (28.3%)
IB	125 (35.4%)
IIA	22 (6.2%)
IIB	49 (13.9%)
IIIA	57(16.1%)
Adjuvant treatment	
No	216 (61.2%)
Yes	119 (33.7%)
Unknown	18 (5.1%)
Neoadjuvant treatment	
No	298 (84.4%)
Yes	52 (14.7%)
Unknown	3 (0.8%)

* Smoking history was assigned based on the CDC definitions (Accessed on Jun 29, 2010. http://www.cdc.gov/nchs/nhis/tobacco/tobacco_glossary.htm)

Never smoker: An adult who has never smoked, or who has smoked less than 100 cigarettes in his or her lifetime. Former smoker: An adult who has smoked at least 100 cigarettes in his or her lifetime but who had quit smoking at the time of interview. Current smoker: An adult who has smoked 100 cigarettes in his or her lifetime and who currently smokes cigarettes.

† According to the American Joint Committee on Cancer Staging Manual 6th edition.

### Recurrence-free survival and overall survival

We further correlated the expression levels of IGF-1R and pIGF-1R with clinicopathological characteristics and patient survival. The median follow-up times were 6.8 and 5.3 years for the TMA I and TMA II datasets, respectively. The expression levels of IGF-1R and pIGF-1R/IR were evaluated as continuous variables using the Cox Proportional Hazard model. The univariate analysis of the hazard ratios of the biomarkers for OS and RFS are summarized in Table 3 (TMA I) and Table 4 (TMA II). Among the baseline clinical characteristics, the univariate analyses revealed that female sex (only in TMA II), and early cancer stage were significantly associated with improved OS (Tables 3 and 4). In addition, the analyses also revealed that increased cytoplasmic IGF-1R expression was associated with decreased RFS in NSCLC patients (only in TMA II) (Table 4). After adjusting for age, gender, histology and stage, a multivariate analysis was performed to determine whether these biomarker expression levels were independent prognostic factors in this population. Neither IGF-1R nor pIGF-1R expression was a significant marker for OS and RFS in the multivariate analysis (data not shown).

**Table 3:** Univariate analyses of clinical prognostic factors and biomarkers (TMA I)

Variable	HR for OS (95% CI)	p-value	HR for RFS (95% CI)	p-value*
Age	.997 (.979-1.015)	.723	1.040 (1.025-1.056)	<.0001
Gender (M vs.F)	.813 (.549-1.203)	.300	1.324 (.981-1.786)	.066
Race (white vs. other)	2.123 (1.261-3.574)	.005	1.043 (.640-1.699)	.867
Smoker (Ever vs. never)	.669 (418-1.072)	.095	1.136 (.754-1.712)	.542
Histology (ADC vs. SCC)	.946 (.626-1.430)	.792	1.442 (1.068-1.949)	.017
Stage (vs. I)				
II	2.005 (1.201-3346)	.008	1.566 (1.069-2.293)	.021
III/IV	4.106 (2.557-6.593)	<.001	2.707 (1.365-5.370)	.004
IGF-1R				
cytoplasmic	.998 (.993-1.003)	.381	1.000 (.997-1.003)	.961
membrane	.995 (.988-1.003)	.238	1.000 (.995-1.005)	.962
p-IGF-IR/1R				
cytoplasmic	1.008 (.987-1.030)	.444	1.011 (.996-1.026)	.141
membrane	1.004 (.999-1.009)	.157	1.004 (.999-1.008)	.099

* Univariate p-values are based on Cox Proportional Hazard Model.

Abbreviation: HR – hazard ratio, OS – overall survival, RFS – recurrence-free survival, CI – confidence interval, ADC – adenocarcinoma, SCC – squamous cell carcinoma, IR – insulin receptor and IGF-1R – insulin-like growth factor 1 receptor.

**Table 4:** Univariate analyses of clinical prognostic factors and biomarkers (TMA II)

Variable	HR for OS (95% CI)	p-value	HR for RFS (95% CI)	p-value*
Age	1.015 (0.998-1.031)	0.08	1.007 (0.993-1.022)	0.31
Gender (M vs.F)	1.468 (1.067-2.020)	0.0186	1.317 (0.997-1.740)	0.053
Race (white vs. other)	0.747 (0.457-1.222)	0.245	0.784 (0.504-1.222)	0.28
Smoker				
Former vs. Never	1.045 (0.597-1.828)	0.88	1.081 (0.667-1.751)	0.75
Current vs. Never	1.219 (0.698-2.129)	0.49	1.108 (0.681-1.803)	0.68
Histology (ADC vs. SCC)	0.831 (0.600-1.151)	0.27	0.809 (0.609-1.074)	0.14
Stage (vs. I)				
II	1.608 (1.090-2.374)	0.0157	1.73 (1.226-2.440)	0.0018
IIIA	2.399 (1.613-3.570)	<0.0001	2.734 (1.930-3.872)	<0.0001
IGF-IR				
cytoplasmic	1.003 (0.999-1.007)	0.19	1.004 (1.000-1.008)	0.034
membrane	1.002 (0.992-1.011)	0.70	1.001 (0.992-1.010)	0.88
p-IGF-112/IR				
cytoplasmic	0.997 (0.985-1.010)	0.50	0.990 (0.978-1.003)	0.12
membrane	0.998 (0.993-1.003)	0.41	0.997 0.992-1.001	0.16

* Univariate *p*-values are based on Cox Proportional Hazard Model

Abbreviations: HR = Hazard Ratio, CI = Confidence interval, OS = overall survival, RFS: relapse-free survival.

### TCGA data analysis on IGF-1R mRNA and protein expression

**Figure 3:**
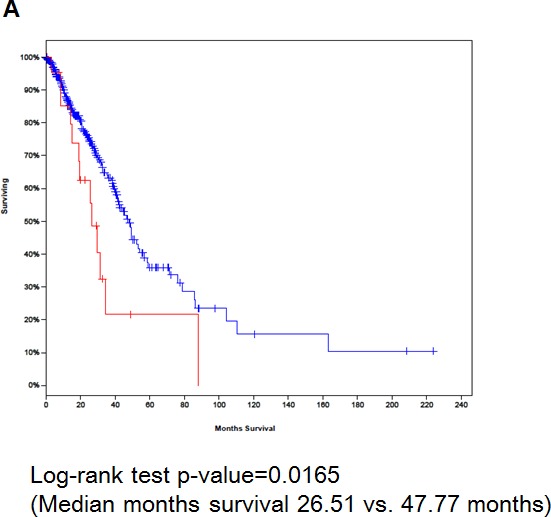

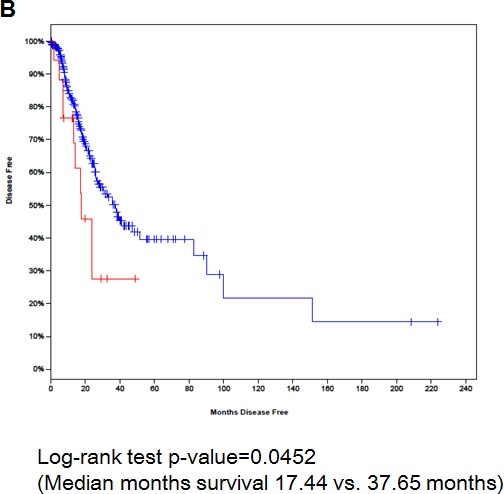
Kaplan–Meier overall survival (OS) and disease-free survival (DFS) curves for ADC patients with (red line) or without (blue line) increases in IGF-1R mRNA expression based on TCGA biospecimens and clinical data (**A**) OS and (**B**) DFS.

A total of three lung cancer studies were identified from the TCGA Research Network portal including TCGA provisional data, TCGA Nature 2012, and TCGA Nature 2014 data [15, 16] for IGF-1R mRNA and protein expression. No information was available for pIGF-1R mRNA or protein expression from the database. In general, upregulation of IGF-1R mRNA was reported for both ADC and SCC, ranging from 5.3% to 6.7%. Significant differences in OS (Fig. 3A) and DFS (Fig. 3B) were observed in a study including ADC patients; the median survival time was significantly shorter for cases with increased IGF-1R mRNA expression than for those without changes (OS median survival 26.51 vs. 47.77 months, *P* = 0.017; DFS median survival 17.44 vs. 37.65 months, *P* = 0.045). Alteration in IGF-1R protein expression was identified in 4.6% of cases with SCC from TCGA provisional data (*N* = 195). However, the changes were not associated with statistically significant changes in survival. No changes in protein expression were identified from cases with ADC.

## DISCUSSION

In this study, we performed an IHC analysis of the expression patterns of IGF-1R and pIGF-1R in patients with NSCLC using two large independent TMAs and publicly available query data known as the TCGA Research Network data accessible from the cBioPortal [15, 16]. Findings from our study indicated the prognostic value of IGF-1R mRNA expression in patients with ADC only from the TCGA data. The clinical development of IGF-1R inhibitors is ongoing [5, 17]. To select patients who would be most likely to benefit from these agents, an analysis of target protein expression and the identification of a specific population are required. Given the limited amount of preclinical data on expression patterns or predictors of responses to anti-IGF-1R, antisense RNA, or microRNA, research findings such as those presented in this study are needed to provide insight into the potential prognostic values of these biomarkers.

The IGF-1R pathway plays a critical role in tumorigenesis, angiogenesis, metastasis, and resistance to existing forms of anti-cancer therapy. Accordingly, a variety of strategies to target IGF-1R have been in preclinical or clinical stages for the treatment of cancer patients. However, the mechanisms causing their overexpression in NSCLC are not clear. Inappropriate expression of growth factors or their receptors causes a decrease in the requirement for exogenous growth factors to support the growth and progression of tumors [18]. Dysregulated activation of the IGF-1R pathway by ectopic overexpression of IGF-1R in murine hemopoietic cells causes these cells to gain independence from growth factor requirements and have an increase in resistance to apoptosis [19]. An aberrant regulation of IGF-1R enables MIA PaCa-2 pancreatic cancer cells to escape rapidly from quiescence, makes them growth factor independent, and promotes survival during unfavorable conditions [20].

In lung cancer, elevated plasma levels of IGF-1 have been associated with an increased risk of the disease [21]. Conversely, high plasma levels of insulin-like growth factor binding protein 3 (IGFBP-3) have been associated with a reduced risk [22]. We have previously demonstrated loss of IGFBP-3 expression in early-stage NSCLC [23]. We further reported that hypermethylation of the IGFBP-3 promoter is a frequent abnormality in NSCLC, which is one mechanism that silences IGFBP-3 expression in lung cancers, particularly NSCLC [24]. Recently, we showed that the overexpression of IGF-1 predicts poor survival among patients with NSCLC, especially those with ADC [25]. The main components of the IGF axis also include IGF-1R and its highly structurally conserved family member, insulin receptor (IR). Overexpression of IGF-1R has been found in NSCLC cell lines [26]. A previous study implied that high IGF-1R expression is associated with poor survival in surgically treated NSCLC patients [27]. We have also shown high levels of IGF-1R and IR expression in NSCLC [28]. In this study, we observed the impact of IR as an independent predictive marker of NSCLC survival. However, IGF-1R expression did not have significant impact on RFS and OS of the patients. Similarly, previous studies do not consistently support a definite impact of IGF-1R protein expression on survival of NSCLC [6, 7, 11].

Based on these controversial findings, we investigated patterns of IGF-1R expression and their association with patient survival. Although previous studies with a relatively small sample size did not identify an association of IGF-1R protein expression with histology in NSCLC samples [18, 29], recent reports have demonstrated an association between high IGF-1R expression and squamous histology [7, 11, 12, 19]. Consistent with the previous findings, we observed a significant association between high IGF-1R expression and squamous histology in the present study. However, no significant difference was found in survival between the high-IGF-1R-expression and low-expression groups even though various exploratory cut-off values were employed.

A second level of regulation of IGF-1R expression is upregulation of *IGF-1R* mRNA expression. The IGF-1R gene copy number was evaluated in previous studies. Fluorescent in situ hybridization (FISH) analysis revealed that the IGF-1R gene copy number was increased in a substantial proportion of NSCLC samples (27%, using criteria developed for *EGFR* gene copy number assessment) [12]. A high *IGF-1R* gene copy number has positive prognostic value in NSCLC patients in the study [11]. Consistent with the results from the previous study, our TCGA data analysis revealed increased mRNA levels in NSCLC patients. Moreover, increased levels of mRNA were significantly associated with reductions in OS and DFS of NSCLC patients, especially those with ADC. This suggested that, possibly, the stability of IGF-1R mRNA transcripts in NSCLC cells is regulated by some factor(s) present in the cells. A previous study suggested that stabilization of IGF-1R mRNA via a lack of the unidentified factors that degrade IGF-1R mRNA contributes to aberrant and constitutive activation of this receptor [20].

In conclusion, using large-scale TMAs and TCGA data, we have determined the expression patterns of IGF-1R and pIGF-1R and their association with clinicopathological characteristics. We show that pIGF-1R and IGF-1R expression was higher in patients with SCC than in those with ADC. In addition, we show the lack of prognostic impact of these biomarkers on survival in patients with NSCLC. In contrast, high levels of IGF-1R mRNA appeared to predict decreased survival of patients with NSCLC. IGF-1R overexpression has been suggested to serve as a predictive biomarker of the response to anti-IGF-1R antibodies, such as R1507 [21] and figitumumab [22]. Previous studies also interrogated *IGF-1R* gene copy number status in a panel of NSCLC cell lines and found cell lines with high *IGF-1R* copy number were significantly more sensitive to the *IGF-1R* antibody R1507 compared with those with low *IGF-1R* copy number [7], suggesting the potential value of IGF-1R gene copy number as a predictive marker for anti-IGF-1R monoclonal antibody. These previous results and our current findings suggest that the IGF-1R mRNA level could be a useful predictive biomarker in identifying subsets of NSCLC patients who may be candidates for future IGF-1R inhibitor-based clinical trials.

## PATIENTS AND METHODS

### Patient characteristics

The patient baseline characteristics from the two tissue microarray (TMA) sets are presented in Tables 1 and 2. Detailed clinical and pathological information were available for most of these cases and included patients' demographic data, smoking history (never smokers or ever smokers, patients who had smoked at least 100 cigarettes in their lifetime), pathologic tumor-node-metastasis (TNM) staging, OS time, and recurrence-free survival (RFS) time. Gender was well balanced in both of the datasets, and adenocarcinoma (ADC) patients represented nearly two-thirds of each dataset. Most patients had early-stage lung cancer and were former or current smokers. The tissue banking and research conduct were approved by the M.D. Anderson Cancer Center Institutional Review Board. All patients provided informed consent, and any personal identifiers were removed from the database.

### Case selection and TMA construction

We obtained archived formalin-fixed and paraffin-embedded samples from previously described tissue banks at The University of Texas M.D. Anderson Cancer Center (Houston, TX) [23]. The tissue specimens were originally collected between 1997 and 2005 and were classified using the 2004 World Health Organization classification system [25]. We selected specimens to construct two TMA sets. TMA set I comprised NSCLC specimens obtained from patients who underwent surgery at the M.D. Anderson Cancer Center in 1997–2003, and TMA set II comprised NSCLC tumor specimens obtained from patients who underwent surgery at the same institution in 2003–2005. Only patients with available staging information were included in the analyses (*N* = 352 for TMA I and *N* = 353 for TMA II). To clarify the impact of biomarker expression on resectable NSCLC survival, patients with advanced stages, i.e., stages IIIB and IV, or patients in early stages who received a suboptimal surgery, such as wedge resection, were excluded from TMA II in the survival analysis (*N* = 270). After histological examination of the NSCLC specimens, the NSCLC TMAs were constructed by removing three 1 mm-diameter cores from each tumor at three different sites (periphery, intermediate and central tumor sites). The TMAs were prepared with a manual tissue arrayer (Advanced Tissue Arrayer ATA100, Chemicon International, Temecula, CA).

### Immunohistochemistry and analysis

We previously observed significantly higher levels of IGF-1R in SCC specimens than in ADC specimens [26]. Thus, we investigated the activation of the IGF-1R pathway in NSCLC as well as the expression of IGF-1R and pIGF-1R by performing immunohistochemical (IHC) analysis using TMA I and TMA II, which were associated with median follow-up times of 6.8 and 5.3 years, respectively. The primary antibodies for IHC analysis were purchased from Cell Signal Technology (IGF-1R and pIGF-1R). For pIGF-1R expression in the tissue samples, we used antibodies against pIGF-1R (Tyr 1135/1136)/pIR, Tyr1162/1163) employed previously [26].

Details of the IHC protocol have been described previously [23]. Expression was quantified by two independent observers (C.B. and I.I.W.). For each patient, the results from three cores were averaged. The results for each observer were averaged to obtain the final IHC score. Cytoplasmic, membranous and nuclear expression were quantified using a four-value intensity score (0, 1+, 2+, and 3+) and the percentage (0–100%) of the extent of reactivity. Next, the expression score was obtained by multiplying the intensity score and reactivity extension values (range, 0–300). A tumor sample was considered positive if the score was above the median and negative otherwise.

### IGF-1R mRNA and protein expression and survival from The Cancer Genome Atlas (TCGA) data

We searched the TCGA data portal to identify alterations in IGF-1R mRNA and protein expression, and the relationships between changes in the expression and OS or disease-free survival (DFS) were evaluated. The TCGA Research Network portal was used for evaluating alterations in mRNA and protein expression, and clinical data for comparing survival; these are available at www.cbioportal.org/public-portal [27, 28].

### Statistical analysis

The summary statistics for the biomarker expression levels according to patient baseline characteristics were computed. The Wilcoxon rank sum test and Kruskal–Wallis test were used to compare biomarker expression among different subgroups defined by categorical variables, such as histology. The OS and RFS of each subgroup of patients were determined by the Kaplan–Meier method and compared using the log-rank test. Cox proportional hazard models were used for multivariate analyses. All statistical tests were two-sided, and *P* ≤ 0.05 was considered significant. The association between changes in IGF-1R mRNA expression and OS or DFS was evaluated by a log-rank test and provided by the TCGA Research Network [27, 28].

## References

[1] Jemal A, Bray F, Center MM, Ferlay J, Ward E, Forman D (2011). Global cancer statistics. CA: a cancer journal for clinicians.

[2] Siegel R, Naishadham D, Jemal A (2013). Cancer statistics, 2013. CA: a cancer journal for clinicians.

[3] Keith RL, Miller YE (2013). Lung cancer chemoprevention: current status and future prospects. Nature reviews Clinical oncology.

[4] Johnson DH, Schiller JH, Bunn PA (2014). Recent clinical advances in lung cancer management. Journal of clinical oncology : official journal of the American Society of Clinical Oncology.

[5] Tao Y, Pinzi V, Bourhis J, Deutsch E (2007). Mechanisms of disease: signaling of the insulin-like growth factor 1 receptor pathway--therapeutic perspectives in cancer. Nature clinical practice Oncology.

[6] Dziadziuszko R, Camidge DR, Hirsch FR (2008). The insulin-like growth factor pathway in lung cancer. Journal of thoracic oncology : official publication of the International Association for the Study of Lung Cancer.

[7] Gong Y, Yao E, Shen R, Goel A, Arcila M, Teruya-Feldstein J, Zakowski MF, Frankel S, Peifer M, Thomas RK, Ladanyi M, Pao W (2009). High expression levels of total IGF-1R and sensitivity of NSCLC cells in vitro to an anti-IGF-1R antibody (R1507). Plos One.

[8] Kim YH, Sumiyoshi S, Hashimoto S, Masago K, Togashi Y, Sakamori Y, Okuda C, Mio T, Mishima M (2012). Expressions of insulin-like growth factor receptor-1 and insulin-like growth factor binding protein 3 in advanced non-small-cell lung cancer. Clinical lung cancer.

[9] Karp DD, Paz-Ares LG, Novello S, Haluska P, Garland L, Cardenal F, Blakely LJ, Eisenberg PD, Langer CJ, Blumenschein G, Johnson FM, Green S, Gualberto A (2009). Phase II study of the anti-insulin-like growth factor type 1 receptor antibody CP-751,871 in combination with paclitaxel and carboplatin in previously untreated, locally advanced, or metastatic non-small-cell lung cancer. Journal of clinical oncology : official journal of the American Society of Clinical Oncology.

[10] Langer CJ, Novello S, Park K, Krzakowski M, Karp DD, Mok T, Benner RJ, Scranton JR, Olszanski AJ, Jassem J (2014). Randomized, phase III trial of first-line figitumumab in combination with paclitaxel and carboplatin versus paclitaxel and carboplatin alone in patients with advanced non-small-cell lung cancer. Journal of clinical oncology : official journal of the American Society of Clinical Oncology.

[11] Cappuzzo F, Tallini G, Finocchiaro G, Wilson RS, Ligorio C, Giordano L, Toschi L, Incarbone M, Cavina R, Terracciano L, Roncalli M, Alloisio M, Varella-Garcia M, Franklin WA, Santoro A (2010). Insulin-like growth factor receptor 1 (IGF1R) expression and survival in surgically resected non-small-cell lung cancer (NSCLC) patients. Annals of oncology : official journal of the European Society for Medical Oncology / ESMO.

[12] Dziadziuszko R, Merrick DT, Witta SE, Mendoza AD, Szostakiewicz B, Szymanowska A, Rzyman W, Dziadziuszko K, Jassem J, Bunn PA, Varella-Garcia M, Hirsch FR (2010). Insulin-like growth factor receptor 1 (IGF1R) gene copy number is associated with survival in operable non-small-cell lung cancer: a comparison between IGF1R fluorescent in situ hybridization, protein expression, and mRNA expression. Journal of clinical oncology : official journal of the American Society of Clinical Oncology.

[13] Kikuchi R, Sonobe M, Kobayashi M, Ishikawa M, Kitamura J, Nakayama E, Menju T, Miyahara R, Huang CL, Date H (2012). Expression of IGF1R is associated with tumor differentiation and survival in patients with lung adenocarcinoma. Annals of surgical oncology.

[14] Fidler MJ, Shersher DD, Borgia JA, Bonomi P (2012). Targeting the insulin-like growth factor receptor pathway in lung cancer: problems and pitfalls. Therapeutic advances in medical oncology.

[15] Cancer Genome Atlas Research N (2014). Comprehensive molecular profiling of lung adenocarcinoma. Nature.

[16] Cancer Genome Atlas Research N (2012). Comprehensive genomic characterization of squamous cell lung cancers. Nature.

[17] Kim LC, Song L, Haura EB (2009). Src kinases as therapeutic targets for cancer. Nature reviews Clinical oncology.

[18] Ludovini V, Bellezza G, Pistola L, Bianconi F, Di Carlo L, Sidoni A, Semeraro A, Del Sordo R, Tofanetti FR, Mameli MG, Daddi G, Cavaliere A, Tonato M, Crino L (2009). High coexpression of both insulin-like growth factor receptor-1 (IGFR-1) and epidermal growth factor receptor (EGFR) is associated with shorter disease-free survival in resected non-small-cell lung cancer patients. Annals of oncology : official journal of the European Society for Medical Oncology / ESMO.

[19] Reinmuth N, Kloos S, Warth A, Risch A, Muley T, Hoffmann H, Thomas M, Meister M (2014). Insulin-like growth factor 1 pathway mutations and protein expression in resected non-small cell lung cancer. Human pathology.

[20] Nair PN, De Armond DT, Adamo ML, Strodel WE, Freeman JW (2001). Aberrant expression and activation of insulin-like growth factor-1 receptor (IGF-1R) are mediated by an induction of IGF-1R promoter activity and stabilization of IGF-1R mRNA and contributes to growth factor independence and increased survival of the pancreatic cancer cell line MIA PaCa-2. Oncogene.

[21] Arbet-Engels C, Tartare-Deckert S, Eckhart W (1999). C-terminal Src kinase associates with ligand-stimulated insulin-like growth factor-I receptor. The Journal of biological chemistry.

[22] Gualberto A, Dolled-Filhart MP, Hixon ML, Christensen J, Rimm DL, Lee AV, Wang Y, Pollak M, Paz-Ares LG, Karp DD (2009). Molecular bases for sensitivity to figitumumab (CP-751,871) in NSCLC. Journal of Clinical Oncology.

[23] Yuan P, Kadara H, Behrens C, Tang X, Woods D, Solis LM, Huang J, Spinola M, Dong W, Yin G, Fujimoto J, Kim E, Xie Y, Girard L, Moran C, Hong WK (2010). Sex determining region Y-Box 2 (SOX2) is a potential cell-lineage gene highly expressed in the pathogenesis of squamous cell carcinomas of the lung. Plos One.

[24] Chang YS, Wang L, Suh YA, Mao L, Karpen SJ, Khuri FR, Hong WK, Lee HY (2004). Mechanisms underlying lack of insulin-like growth factor-binding protein-3 expression in non-small-cell lung cancer. Oncogene.

[25] Brambilla E, Travis WD, Colby TV, Corrin B, Shimosato Y (2001). The new World Health Organization classification of lung tumours. The European respiratory journal.

[26] Kim JS, Kim ES, Liu D, Lee JJ, Solis L, Behrens C, Lippman SM, Hong WK, Wistuba, Lee HY (2012). Prognostic impact of insulin receptor expression on survival of patients with nonsmall cell lung cancer. Cancer.

[27] Gao J, Aksoy BA, Dogrusoz U, Dresdner G, Gross B, Sumer SO, Sun Y, Jacobsen A, Sinha R, Larsson E, Cerami E, Sander C, Schultz N (2013). Integrative analysis of complex cancer genomics and clinical profiles using the cBioPortal. Science signaling.

[28] Cerami E, Gao J, Dogrusoz U, Gross BE, Sumer SO, Aksoy BA, Jacobsen A, Byrne CJ, Heuer ML, Larsson E, Antipin Y, Reva B, Goldberg AP, Sander C, Schultz N (2012). The cBio cancer genomics portal: an open platform for exploring multidimensional cancer genomics data. Cancer discovery.

[29] Cappuzzo F, Toschi L, Tallini G, Ceresoli GL, Domenichini I, Bartolini S, Finocchiaro G, Magrini E, Metro G, Cancellieri A, Trisolini R, Crino L, Bunn PA, Santoro A, Franklin WA, Varella-Garcia M (2006). Insulin-like growth factor receptor 1 (IGFR-1) is significantly associated with longer survival in non-small-cell lung cancer patients treated with gefitinib. Annals of oncology : official journal of the European Society for Medical Oncology / ESMO.

